# The effects of computed tomography image characteristics and knot spacing on the spatial accuracy of B-spline deformable image registration in the head and neck geometry

**DOI:** 10.1186/1748-717X-9-169

**Published:** 2014-07-29

**Authors:** Charlotte L Brouwer, Roel GJ Kierkels, Aart A van ’t Veld, Nanna M Sijtsema, Harm Meertens

**Affiliations:** 1 University of Groningen, University Medical Center Groningen, Department of Radiation Oncology, Groningen, the Netherlands; 2 Department of Radiation Oncology, University Medical Center Groningen, PO Box 30001, Groningen 9700 RB, The Netherlands

**Keywords:** Deformable image registration, B-spline transformation model, Spatial accuracy, B-spline knot spacing, Contrast-to-noise ratio, Head-and-neck, Computed tomography

## Abstract

**Objectives:**

To explore the effects of computed tomography (CT) image characteristics and B-spline knot spacing (BKS) on the spatial accuracy of a B-spline deformable image registration (DIR) in the head-and-neck geometry.

**Methods:**

The effect of image feature content, image contrast, noise, and BKS on the spatial accuracy of a B-spline DIR was studied. Phantom images were created with varying feature content and varying contrast-to-noise ratio (CNR), and deformed using a known smooth B-spline deformation. Subsequently, the deformed images were repeatedly registered with the original images using different BKSs. The quality of the DIR was expressed as the mean residual displacement (MRD) between the known imposed deformation and the result of the B-spline DIR.

Finally, for three patients, head-and-neck planning CT scans were deformed with a realistic deformation field derived from a rescan CT of the same patient, resulting in a simulated deformed image and an *a-priori* known deformation field. Hence, a B-spline DIR was performed between the simulated image and the planning CT at different BKSs. Similar to the phantom cases, the DIR accuracy was evaluated by means of MRD.

**Results:**

In total, 162 phantom registrations were performed with varying CNR and BKSs. MRD-values < 1.0 mm were observed with a BKS between 10–20 mm for image contrast ≥ ± 250 HU and noise < ± 200 HU. Decreasing the image feature content resulted in increased MRD-values at all BKSs. Using BKS = 15 mm for the three clinical cases resulted in an average MRD < 1.0 mm.

**Conclusions:**

For synthetically generated phantoms and three real CT cases the highest DIR accuracy was obtained for a BKS between 10–20 mm. The accuracy decreased with decreasing image feature content, decreasing image contrast, and higher noise levels. Our results indicate that DIR accuracy in clinical CT images (typical noise levels < ± 100 HU) will not be effected by the amount of image noise.

## Background

In radiotherapy, multiple volumetric images of a patient are often acquired to prepare and deliver a treatment plan with high accuracy in terms of dose and position. Nowadays, treatment planning relies on computed tomography (CT) images whether or not in combination with positron emission tomography images and magnetic resonance imaging (MRI). Furthermore, the course of the treatment can be monitored using one or more of these imaging modalities. In-room cone-beam CT (CBCT) images are often acquired for treatment position verification. These CBCTs may potentially be used for treatment evaluation and adaptation. For head-and-neck cancer patients, significant changes in the patient anatomy between the reference situation (e.g. planning CT) and the course of treatment can gradually occur and are mainly related to weight loss, tumor regression and resolution of edema [1,2]. As the head-and-neck anatomy is characterized by several vulnerable normal tissues, often in the vicinity of steep dose gradients, monitoring of these anatomical changes becomes increasingly important. Adaptive radiotherapy protocols are used to correct for anatomic and morphologic changes during treatment, aiming at improved local tumor control and/or a reduction of radiation induced side effects.

Deformable image registration (DIR) algorithms provide a one-to-one spatial mapping of voxels in one image to voxels in the other image. When an adaptive treatment protocol is used, the role of DIR becomes increasingly important, e.g. for contour warping and fractional dose accumulations using the CBCTs and rescan CTs. To approximate the actual given dose in the patient, accurate correlations of anatomical points and (sub)volumes between image sets acquired during treatment are required.

Systems for DIR become more and more (commercially) available, but are not yet widely disseminated and used in treatment planning systems, partly due to the computational costs and the difficulty of validating the results. Nevertheless, an increasing number of publications on clinical applications of DIR in radiotherapy indicate the high expectations of this technique [2-8]. In literature, several parametric deformable transformation models have been proposed [9-11]. Promising characteristics have been ascribed to the basic spline model, B-splines [12].

DIR algorithms are usually governed by a set of configuration parameters. Therefore, prior to application, the following question must be answered: to what extent do these parameters influence the accuracy of the DIR outcome? One of the tunable configuration parameters which determines the quality of a deformation in a B-spline registration, is the B-spline knot spacing (BKS) [13]. A large number of knots resolves locally sharp features, while fewer knots allow for modeling smoother and larger features. The quality of a deformation field from an intensity-based DIR algorithm like the B-splines algorithm also highly depends on the available local feature content in an image. Feature content is associated with the image properties contrast and noise, and is variable among scans of different anatomical sites, and the used scanner (e.g. CT or CBCT) and scanner settings.

The number of studies that have paid attention to the effect of DIR configuration parameters and image characteristics is limited. The effect of image noise [14], intensity gradients (contrast and feature content) [15], and B-spline knot placement [11,13,15] on the accuracy of a deformation has been investigated previously. Murphy *et al*. demonstrated that CT image noise caused no significant loss of registration accuracy [14]. The authors evaluated the registration quality by contour comparisons using contour distances. Contours are generally defined at the border of anatomical structures containing features with sufficient image contrast. At these regions, intensity-based DIR algorithms generally perform better than within more homogeneous regions. Within a contour, however, the DIR quality cannot be evaluated with these contour-based evaluation methods, limiting its use for dose accumulations. The effect of image noise on registration accuracy has been studied so far only using contour distances. To our knowledge, no studies determined the registration accuracy on a voxel to voxel basis. Furthermore, it would be worthwhile to study the combination of the effect of image characteristics (i.e. image noise, contrast, and feature content) and B-spline knot placement on the registration accuracy. Zhong *et al.*[15] previously suggested that the number of B-spline knots for an optimal registration is affected by the intensity gradients of the underlying images.

The purpose of this simulation study was to quantitatively evaluate the effect of uniform B-spline knot spacings and image characteristics on the accuracy of a free-form deformable registration using B-splines. To assess the quality of the deformation field, a known deformation was applied to synthetically generated CT images and real CT volumes of the head-and-neck region. With these simulated deformed images, the ability of the algorithm to recover the *a-priori* known deformation was investigated on a voxel to voxel basis.

## Methods

In this simulation study we included synthetically generated (fixed) phantom images with varying image characteristics. For each fixed phantom image, a corresponding simulated moving image was created with a smooth, Gaussian shaped, B-spline deformation. Furthermore, planning CT (fixed) images with corresponding simulated moving images of three head-and-neck cancer patients were included.

### Phantom data

**Figure 1:**
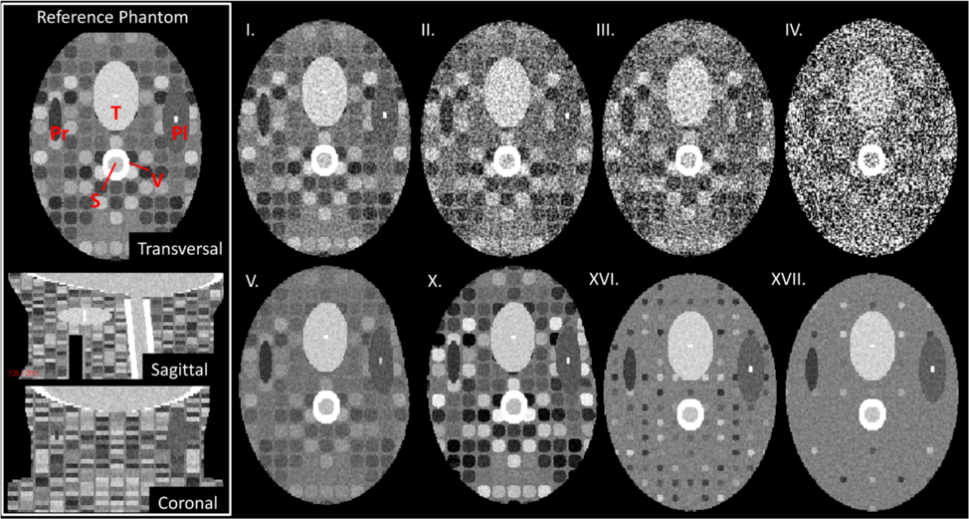
**A selection of the synthetically generated “head-and-neck” phantoms as used in this study.** A transversal, sagittal and coronal cross-section of the reference phantom is shown within the white box. Abbreviations of the simulated structures: T = tumour; V = vertebrae; Pl = parotid left; Pr = parotid right; S = spinal cord. Phantoms I-IV have similar properties as the reference phantom but with varying noise levels (1SD noise = ± 40, ± 80, ± 100, and ± 200 HU, respectively). Simulated deformed phantom images are shown by V and X (with grey-value factor of 0.5 and 1.5, respectively). Phantoms XVI and XVII vary in disc spacing (7.0 and 19.0 mm, respectively) from the reference situation (1.0 mm). The properties of all phantoms are listed in Table 1.

Multiple synthesized phantom CT images *I*_*F*_*(****x****)* and *I*_*M*_*(****x****)* simulating the head-and-neck region were created with varying image contrast, noise and feature content. Figure 1 shows a transversal, sagittal and coronal cross-section of the reference phantom. The phantoms consisted of 60 slices, a FOV of 321 × 251 mm, an in-plane voxel size of 1.00 × 1.00 mm, and a slice thickness of 2.00 mm. Other characteristics of the reference phantom are listed in Table 1. From the reference phantom, seventeen more phantoms were created with varying amounts of image contrast, image noise, and varying disc spacing (phantom properties in Table 1). The discs were added to provide the intensity-based DIR algorithm with varying local feature content. For different phantoms, the image contrast was adapted by multiplying the discs grey values by a grey value factor (GF). The GFs were chosen such that the resulting images included contrast levels comparable to clinical cases of head-and-neck CT images. Transversal slices of the most important phantoms are shown in Figure 1.

**Table 1:** Phantom characteristics

**Reference phantom properties:**															
Simulated structure		Density (HU)			Dimensions [AP, LR, SI] (mm)					Shape					
Body		0.0			220 × 150 × 120										
Parotid gland left (Pl)		-100			60 × 24 × 50					Ellipsoidal					
Parotid gland right (Pr)		-200			40 × 12 × 30					Ellipsoidal					
Tumor (T)		250			60 × 40 × 20					Ellipsoidal					
Vertebrae (V)		1000			28 × 24					Tubical					
Spinal cord (S)		200			12 × 12					Tubical					
Two bony structures near parotid glands		1000			16 × 12 × 12					Square					
Discs (spacing of 1.0 mm)		-250 - 250			12 × 4.0					Tubical					
**Phantom images:**	**Ref.**	**I**	**II**	**III**	**IV**	**V**	**VI**	**VII**	**VIII**	**IX**	**X**	**XI**	**XII**	**XII**I	**XIV**
Image noise (1SD, ± HU)	20	40	80	100	200	20	40	80	100	200	20	40	80	100	200
Grey value factor discs (GF)	1.0	1.0	1.0	1.0	1.0	0.5	0.5	0.5	0.5	0.5	1.5	1.5	1.5	1.5	1.5
Contrast-to-noise ratio (CNR)	17.5	8.75	4.38	3.50	1.75	11.3	5.63	2.81	2.25	1.13	23.8	11.9	5.94	4.75	2.38
Phantom images:	XV	XVI	XVII												
Diameter discs (mm)	6.0	6.0	6.0												
Spacing discs (mm)	1.0	7.0	19												

Ref. indicates the characteristics of the reference phantom (see Figure 1).

All phantoms were assigned to be fixed images *I*_*F*_*(****x****)*. Subsequently, from each *I*_*F*_*(****x****)* a corresponding simulated moving phantom image *I*_*M*_*(****x****)* was obtained by an intentionally imposed transformation of *I*_*F*_*(****x****).* The imposed deformation was obtained through a transformation *T(****x****)* based on B-splines with a 10 mm knot spacing. The voxel positions in these images were noted as **x** = (x,y,z). The *a-priori* known imposed transformation consisted of a well-defined smooth (Gaussian shaped) B-spline deformation. With this deformation we focused on the effect of parameter configuration of the B-spline algorithm in an assumedly optimal setting for the registration algorithm. This may limit the generality of the results but offers the opportunity to determine the ability of the algorithm to recover the known ground truth deformation. An example of a transformed phantom is shown in Figure 1V and X. The distribution of moduli of displacement vectors for all voxels in *I*_*F*_*(****x****)* that represent the imposed smooth deformation were chosen to be in the same order of magnitude as those observed in clinical cases.

Finally, five levels of noise were added to the images *I*_*M*_*(****x)*** and *I*_*F*_*(****x****)* of the reference phantom (Table 1 and Figure 1 phantom I-IV). The spectral density at various spatial frequencies in the transverse plane was taken from the noise power spectrum for CT images as given by Siewerdsen *et al.*[16]. The shape of the noise spectrum is a characteristic filtered ramp spectrum in which the noise power spectrum increases at low frequencies due to a ramp filter, and rolls off at higher frequencies due to band limiting processes such as blur and interpolation. The noise levels were denoted by the 1SD of the HUs of the voxels in a homogeneous area of an image slice: 1SD = ± 20, ± 40, ± 80, ± 100, ± 200 HU. Furthermore, for all phantoms, the contrast-to-noise ratio (CNR) was derived by the ratio of the intensity difference between the brightest discs and the simulated left parotid structure (Figure 1. Pl) and 1SD of the image noise.

### Patient data

CT image pairs of three head-and-neck cancer patients were included to evaluate more clinically relevant and plausible deformations. The properties of the helical CT scans were as follows: a FOV of 500 mm, an in-plane voxel size of 0.98 × 0.98 mm, a slice thickness of 2.00 mm, and a tube voltage of 120 kVp. The included patients had tumours that originated in the epiglottic (case A, T4N2M0), the supraglottic (case B, T2N0M0), and in an unknown primary tumour location (case C, TXN3M0). Each image pair consisted of a planning CT *I*_*F*_*(****x****)* and a simulated moving image *I*_*M*_*(****x****)*. The latter was created by a realistic B-spline transformation of the planning CT with an *a-priori* known deformation field, derived from a DIR of the planning CT with a rescan CT (with BKS = 10 mm and 500 iterations) of the same patient.

### Hardware and software

For preparation and evaluation of the data, Matlab R2012a (Version 7.14, MathWorks, Natick, MA) was used. Elastix version 4.6 was used for the registrations [17], which were performed on a standard office PC with an Intel Xeon X5550 CPU, 2.67 GHz with 16 GB DDR3 RAM and 64 bits Windows 7 operating system.

### Deformable image registration

In intensity-based DIR, a moving image *I*_*M*_*(****x****)* must be spatially aligned to a fixed image *I*_*F*_*(****x****)*. Both images can be described as continuous functions of intensity values at position **x.** The registration determines a displacement *u(****x****)* that best matches the two images according to a criterion of similarity. The displacement *u(****x****)* spatially relates two images so that the restored image *I*_*M*_*(****T*** *(****x****))* best matches image *I*_*F*_*(****x****)* at every position ***x***. The transformation *T(****x****)* is then defined by *T(****x****) = (****x*** + *u(****x****))*. So the moving image is deformed to fit the fixed image, but the transformation is defined from the fixed to the moving image [18].

In this study, a B-spline free-form deformation model was used [11,12,19]. The B-spline transformation is modeled as a weighted sum of B-spline basis functions, each term weighted by an adjustable control point, placed on a uniform grid of knots. The spacing of the knots (and the corresponding number of control points) in a B-spline registration is an important, user-selectable parameter. In the remainder of this paper, the spacing of the knots will be referred to as B-spline knot spacing (BKS), expressed in mm. There will be an optimal number of control points and knot spacing for any particular image pair, which depends on the local spatial frequency of the image content. With decreasing knot spacing, the registration accuracy generally improves until the knot spacing can resolve all of the details. If one keeps decreasing the knot spacing further, at some point there will not be enough information in the image to constrain all of them, and the registration will become underdetermined and unreliable. The registration parameters as used in this study are listed in Table 2. Except for the BKS, the parameters were taken from Klein *et al.*[20]. The following BKSs (final knot spacings) were used: 5, 6, 8, 10, 15, 20, 25, 30, and 40. The number of iterations was set to 500. This number of iterations has been proven to be large enough for the optimization to converge to an optimal solution (see Additional file 1).

**Table 2:** Parameters used for B-spline registration

**Cost function**	
Metric of the similarity measure	Advanced Mattes mutual information
Number of grey level histogram bins in each resolution level	32
**Transformation**	
Transformation	Cubic B-spline
Knot spacing at the highest resolution levels (mm)	Same spacing for all three dimensions.
	The effect of various spacings was investigated
Knot spacing schedule in resolution levels 1, 2 and 3	4, 2, and 1
**Optimization**	
Optimizer	Adaptive Stochastic Gradient Descent
Maximum number of iterations in each resolution level	Ratio iterations between levels: 1 2 2
	The effect of the number of iterations was investigated
Parameter values for determination step size *a*_*k*_ (mm) of the optimizer for each resolution level	Same values for all three dimensions.
	a = 6400, A = 50, α = 0.60
**Image sampling**	
Spatial samples used to compute the mutual information in each iteration	Randomly off the voxel grid
Number of spatial samples in each iteration	2048
**Hierarchical strategies**	
Number of resolutions levels	3
Fixed image pyramid	Fixed recursive
Moving image pyramid	Moving recursive
Downsampling factor for multi-resolution image data	Gaussian pyramid with factor 2
Downsampling factor for the image pyramid for each resolution level	4 (resolution 1), 2 (resolution 2), 1 (resolution 3)
For multi-grid transformation model	Knot spacing halved every resolution level

### Imposed deformation

In the presented study, an *a-priori* known set of B-spline coefficient vectors φ_*i,j,k*___*imp*_ was used to create an intentionally deformed simulated moving image, with a BKS of 10 mm. In each knot the φ_*i,j,k*_ values were set as follows: first a knot was chosen near the left parotid-like structure, acting as a center point of deformation, to which φ_*i,j,k*_ was set. Then the B-spline coefficients φ_*i,j,k*_ in all other knots were set based on a three dimensional Gaussian function from the center point, and calculated as function of distance to the center point of the deformation. The maximum local imposed displacement in the left parotid-like structure was 10 mm.

In general, the registrations were performed in three steps. First the center point of the field of views of the images were aligned. Second, the images were rigidly registered. In the final step, the free-form DIR was performed. Because the simulated moving images were created from the fixed images using a known deformation field, no rigid registration was required.

### Evaluation of deformable registrations

The deformation vector field (DVF) from the known imposed deformation (DVF_imp_) and corresponding result of the registration (DVF_reg_) were calculated using Transformix [17]. Note that the grid of the deformation field was equal to the image resolution and differs from the BKS. The registration accuracy was quantified by the average difference between DVF_imp_ and DVF_reg_ and expressed as the mean residual displacement (MRD). For the phantom images, the MRD was defined in a region near the initially imposed deformation; the ellipsoidal left parotid structure (Figure 1 Pl).

For the three head-and-neck cancer patient cases, the MRD was defined within the whole patient volume as deformations were simulated throughout the whole volume. Furthermore, the spatial distribution of moduli of DVF_imp_ and DVF_reg_ were demonstrated by histogram comparisons.

## Results

### Phantom data

**Figure 2:**
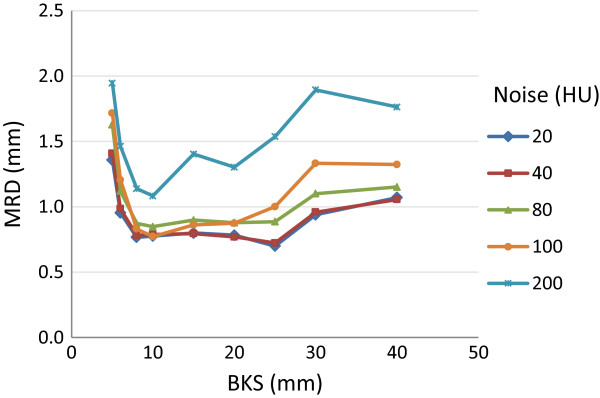
Mean residual displacement (MRD) for different noise levels (±HU) and B-spline knot spacings (BKSs).

For all phantoms the simulated moving image *I*_*M*_*(****x****)* was deformed to the fixed image *I*_*F*_*(****x****)* and the MRD was recorded within the left parotid-like structure*.* The MRD of the phantoms with constant feature content but varying noise levels (i.e. the reference phantom and phantom I-IV as listed in Table 1 and Figure 1) were acquired and compared for registrations with different BKSs (Figure 2). Phantoms with clinically realistic noise levels (1SD ≤ ± 100 HU) which were registered with BKS-values between 8-20 mm, resulted in a MRD < 1.0 mm. The MRD was lowest (0.7 mm) for image sets with a noise level of ± 20 and ± 40 HU (1SD). Only for the phantom with a simulated noise level of ± 200 HU the MRD was > 1.0 mm at all BKSs.

**Figure 3:**
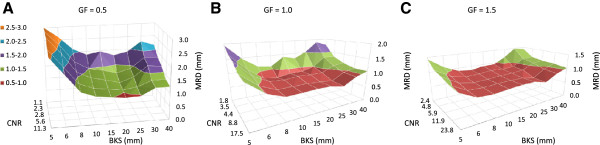
**Mean residual displacement (MRD) for varying contrast-to-noise (CNR) levels and B-spline knot spacings (BKSs).** The image noise varied from 20–200 HU (1 SD) in combination with **(A)** GF = 0.5 (Phantom V-IX), **(B)** GF = 1.0 (Reference phantom and phantom I-IV), and **(C)** GF = 1.5 (Phantom X-XIV). Phantoms according to Table 1. GF = grey value factor. Note that the overall MRD decreased with increasing GF and note the spacing of the BKS-axis.

The intensity of the discs in the reference phantom ranged from ± 250 HU (GF = 1.0). Additionally, phantom images were created with discs intensities of ± 125 (GF = 0.5) and ± 375 HU (GF = 1.5), and with different levels of noise (see phantoms I-XIV in Table 1). From the 15 resulting image sets the CNR was determined. In total, 135 registrations were performed with varying CNR and BKSs (Figure 3). Overall, MRD-values reduced with increasing image contrast. For phantoms with limited contrast (GF = 0.5), MRD-values < 1.0 mm were only observed at CNR = 11.3 and BKS = 20 mm (Figure 3A). For the phantoms with GF ≥ 1.0 the registrations resulted in MRDs < 1.0 mm for CNR ≥ 3.5 and a BKS between 8–25 mm (Figure 3B and C).

**Figure 4:**
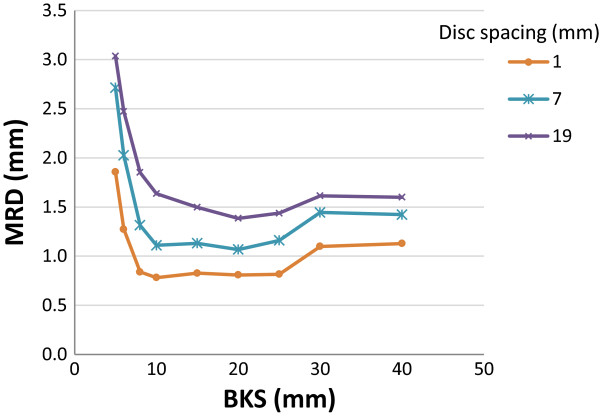
**Registration accuracy for varying image feature content.** The MRD as function of BKS for phantoms with varying disc spacings (phantom XV-XVII) (see phantom properties in Table 1). Abbreviations: MRD = mean residual displacement, BKS = B-spline knot spacing.

**Figure 5:**
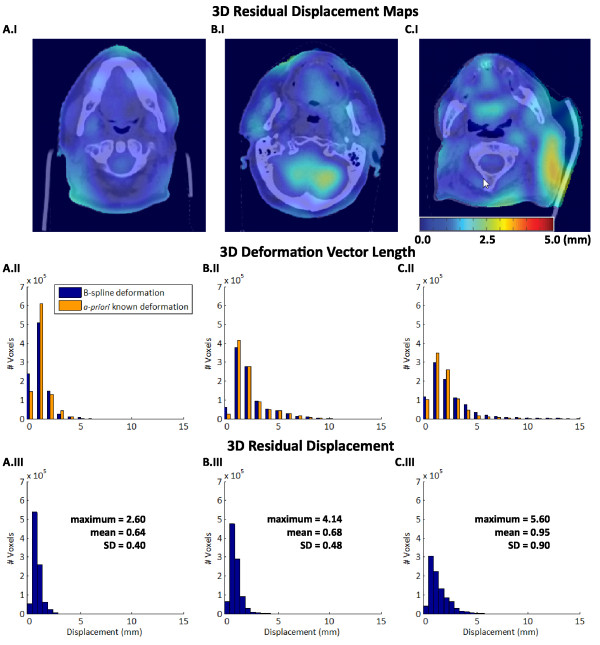
**Registration results of three head-and-neck CT cases (patient A, B, C) with a realistic known imposed displacement derived from a rescan CT of the same patient.** The images in row I show the 3D residual displacement maps between the simulated moving image and the target image, plotted on an axial CT slice of the planning CT. The histograms (row II) indicate the 3D deformation vector length of the known imposed displacements and the resulting registration. The histograms (row III) indicate the 3D residual displacements between known imposed displacements and the resulting registration at BKS = 15 mm.

**Figure 6:**
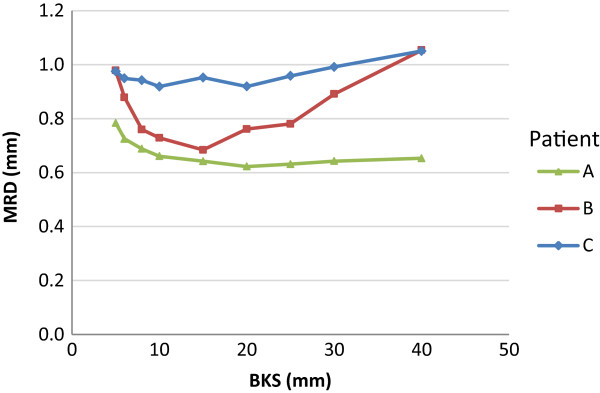
Mean residual displacement (MRD) at different B-spline knot spacings (BKSs) for three head-and-neck CT cases.

To study the effect of varying feature content on the registration accuracy, phantoms with different disc spacing were created (see phantom XV-XVII in Table 1 and Figure 1). The registration of the phantom images with the smallest disc spacing (i.e. 1 mm in between discs) showed similar MRDs for all BKSs (Figure 4). Increased disc spacing (i.e. 7 and 19 mm in between discs; phantom XVI and XVII), resulted in MRDs > 2.0 mm for BKS < 8.0 mm (Figure 4).For three head-and-neck cancer patients a simulated moving image was created using a rescan CT of each individual patient. For each patient, the residual displacement is shown for three representative axial cross-sections throughout the body volume of the planning CT (Figure 5AI-CI). The largest residual displacement was observed near tissue-air transitions and within homogenous regions, such as the brain. Figure 5AII-CII shows the corresponding distribution of the deformation vector length of the known imposed and the resulting registration. The deformations between the planning CT and the rescan CT were of similar magnitude as the artificially imposed Gaussian shaped B-spline deformation to the phantom images. The distribution of the residual displacements after a registration with BKS = 15 mm is shown in Figure 5AIII-CIII. For all patients the MRD was < 1.0 mm.Figure 6 plots the MRD as function of BKS for patient A-C. The registrations resulted in MRDs of < 1.0 mm for all cases, using a BKS < 30 mm. The MRD was lowest using a BKS between 10–20 mm. The neck flexion of patient B was different from patient A and C, and therefore more brain tissue was included in the image of patient B. The brain tissue included higher residual displacements than those in the neck region. The highest MRD for patient C was found in homogeneous tumor tissue.

## Discussion

In this study the effect of image contrast, noise and feature content on a B-spline deformation in synthetically generated phantom images was evaluated at varying B-spline knot spacings. Our results demonstrate that with a BKS between 10 – 20 mm, the lowest MRD (smaller than the size of one image voxel) could be achieved in both the registration of phantom images and real CT scans.

Tanner *et al*. [21] evaluated the DIR accuracy similar to our study. Physically plausible breast deformations of dynamic contrast-enhanced MR images were simulated using finite element methods. Rigid against affine against B-spline based nonrigid (single-resolution, multi-resolution and volume-preserving) registrations were studied. Performance of these configurations was optimized for 5 patients, and tested on another set of 5 patients. The images were most accurately aligned with volume-preserving single-resolution non-rigid registration employing 40 or 20 mm knot spacing. The mean registration error declined from 1.40 to 0.45 mm for the whole breast, and from 1.20 to 0.32 mm for the enhancing lesion. Although the DIR was performed on MR images, similar accuracies as in the current study were reported.

### Imposed deformation

The imposed B-spline deformation was generated from B-splines on a grid of 10 mm knot spacing and contained one particular spectrum of spatial frequencies of the deformation field. For the registration also a B-spline-based transformation was used. Consequently, the results might be expected to give minimal MRD values for BKS values of 10 mm. However, this study was not meant find the optimal parameter values for all clinical situations, but to demonstrate the impact of BKS and noise, contrast, and feature content in the images on the registration result. Deformations given by a certain algorithm will by definition be a model of reality. However, the use of images with known imposed deformations is at the moment the only method to quantify residual errors of a specific deformation in every voxel in patient images, because the local actual deformation of the patient is not known. Additional, independent means to validate registrations are therefore required.

### Noise, contrast, and feature content

The current study showed that images with noise levels (1SD) > ± 100 HU and a CNR < 3.5 resulted in larger MRDs (Figures 2 and 3). Typical observed noise in head-and-neck images, measured in relatively homogeneous areas of the brain of a patient, showed noise values of ± 12 HU for CT and ± 35 HU for CBCT images (1SD). For 4D-CBCT, this value increased to ± 68 HU (liver patient) and ± 76 HU (lung patient) (1SD). This indicates that in clinical (CB)CT-scans the noise would normally only have a minor influence on the outcome of DIR. In case of image regions with little feature content, i.e. the phantoms with a large disc spacing (phantom XVI and XVII), a BKS of 20 mm seemed optimal (Figure 4). The MRD increased with increasing disc spacing, for all BKSs.

Murphy *et al*. [14] also investigated the effect of noise differences on DIR of fan-beam CTs and CTs with simulated cone-beam noise up to ± 120 HU. The accuracy of their B-spline model-based DIR process was assessed by comparing automatically transferred contours of pelvic organs to manual contours on the original CTs. Changes in DIR accuracy due to increased noise were deduced from changes in automatically transferred contours. In line with our results, the investigators found that the addition of noise caused no significant loss of registration accuracy at noise levels equal to or higher than those normally found in CBCT.

A similar study of Zhong *et al*. [15] involved a low-intensity gradient prostate phantom image which was deformed by a modeled deformation based on region-specific material parameters. The authors observed a minimal MRD of 1.6 mm for multi-resolution B-spline DIR. For a CT scan of a lung patient, the authors found minimal MRD of 1.5 mm. However, this minimal MRD dropped to 0.5 mm if the error was averaged within the lung region only. The authors suggested that regions with different image contrast levels can be registered at different accuracies. This was confirmed by our study, in which higher contrast showed a lower MRD (Figure 3). Zhong *et al.*[15] and our results thus suggest that a customization of image registration parameters should be tailored to the specific region: tissue type, image modality, etc.

### B-spline knot spacing

Our results demonstrated that with a BKS between 10–20 mm, the lowest MRD could be achieved. This corresponds to our hypothesis, that with decreasing BKS, at some point the BKS becomes smaller than the spatial frequency (the local density of detail) in the image. Hence, there will not be enough information in the image to constrain all of the control points, and the registration becomes underdetermined and unreliable. On the other hand, at increasing BKS the registration will at some point be unable to capture the sharpness of the deformation, resulting in a higher MRD. The effect of increasing MRD with BKS < 10 and > 25 mm was more pronounced for low CNR (Figure 3), which can be explained by the fact that low CNR deteriorates feature detection.

### DIR validation

In intensity-based DIR approaches, the image similarity measure of the registration is not related to physical space error in a simple way. Therefore, such a similarity measure by itself provides no clue to the user whether the registration has an acceptable accuracy. In most applications, careful visual inspection remains the first and most important validation check available for previously unseen images. Moreover, validation is usually performed by making supplementary measurements post-registration [22].

It is possible to identify corresponding landmarks or regions independently of the registration process and establish how well the registration brings them into alignment [23]. However, in many applications the true point-to-point correspondence might not be known and may not even exist, for instance, due to loss of mass.

Various kinds of consistency measures are used in DIR validation. The simplest and most commonly used measure verifies the inverse consistency in which the registration of *I*_*M*_*(****x****)* to *I*_*T*_*(****x****)* produces the same alignment as *I*_*T*_*(****x****)* to *I*_*M*_*(****x****)*[24]. An extension to this method, the measure of transitivity, utilizes at least three images, and is described in detail by Bender *et al.*[25]. These authors showed that both the inverse consistency and transitivity check were feasible in DIR of head-and-neck CT images.

In the current study, the DIR accuracy was evaluated by calculating a residual error in displacement between a known imposed displacement and the resulting displacement after DIR in a local volume of interest. Such a volume can be selected, for instance, in a region with high dose gradients where the accuracy in position is important for successful treatment monitoring.

### Rigidity penalty

The B-spline transformation model as used in this paper does not take into account the difference in rigidity of various tissue types, such as a parotid gland relative to bony structures (e.g. the mastoid and the mandible condoyle) in the head-and-neck area of a patient. This likely results in unwanted distortions of rigid objects. Using rigidity regularization, involving a penalty term that penalizes deformations of rigid objects, is one method for restricting deformations. Staring *et al*. [26] proposed such a local rigidity penalty term, which has been included in the registration functionality of Elastix [17]. A promising approach would be to quantify the effect of the rigidity term on the MRD in comparison to the standard DIR with the method presented in this paper.

## Conclusion

The accuracy of B-spline deformations of the head-and-neck geometry could be assessed using known deformations in synthetic phantom images and clinical CT scans. For these cases the highest accuracy in the deformations was obtained for BKS between 10–20 mm. The accuracy decreased with decreasing image feature content (i.e. larger disc spacing) and higher noise levels. For clinical CT images, with noise levels 1SD < ± 100 HU, no effect of image noise on the registration accuracy was found. Real CT scans of the head-and-neck region could be registered within an average accuracy < 1 mm.

## Competing interests

The authors declare that they have no competing interests.

## Authors’ contributions

CLB carried out the design of the study, assisted in the analysis of the data and drafted the manuscript. RGJK carried out the analysis of the data, created the figures and drafted the revised manuscript. AA van ‘t V and NMS helped with critical revision of the manuscript. HM carried out the design of the study, the analysis of the data, and drafting the manuscript. All authors read and approved the final manuscript.

## Supplementary Material

Additional file 1 ***Number of Iterations.*** The number of iterations needed to converge the iteration loop to a minimum depends on the deformable image registration algorithm and the type of images. In the current paper, the number of iterations was set to 500 (360 sec in our case), since this number has been proven to be large enough to obtain the optimal registration results. An example can be seen in **Figure S1**, which depicts the mean residual displacement (MRD) as a function of iteration time for different study phantoms and B-spline knot spacing (BKS) = 15 mm. After 500 iterations (360 s), an MRD plateau was reached for all phantoms. **Figure S1.** Mean residual displacement (MRD) as a function of iteration time, for different phantoms (GF = grey-value factor, SD = 1 standard deviation of the image noise (HU)). 360 seconds corresponds to 500 iterations. B-spline knot spacing was set to 15 mm, SD to ± 20 HU (upper graph) and GF to 1.0 (lower graph). Click here for file
