# Supplementary material for: The effects of computed tomography image characteristics and knot spacing on the spatial accuracy of B-spline deformable image registration in the head and neck geometry
Source: Radiat Oncol. 2014 Jul 29;9:169. doi: 10.1186/1748-717X-9-169 (PMC4128373; doi:10.1186/1748-717X-9-169)
Supplement: Additional file 1 — Number of Iterations. The number of iterations needed to converge the iteration loop to a minimum depends on the deformable image registration algorithm and the type of images. In the current paper, the number of iterations was set to 500 (360 sec in our case), since this number has been proven to be large enough to obtain the optimal registration results. An example can be seen in Figure S1, which depicts the mean residual displacement (MRD) as a function of iteration time for different study phantoms and B-spline knot spacing (BKS) = 15 mm. After 500 iterations (360 s), an MRD plateau was reached for all phantoms. Figure S1. Mean residual displacement (MRD) as a function of iteration time, for different phantoms (GF = grey-value factor, SD = 1 standard deviation of the image noise (HU)). 360 seconds corresponds to 500 iterations. B-spline knot spacing was set to 15 mm, SD to ± 20 HU (upper graph) and GF to 1.0 (lower graph). [file 1748-717X-9-169-S1.docx]

**Appendix**

*Number of Iterations*

The number of iterations needed to converge the iteration loop to a minimum depends on the deformable image registration algorithm and the type of images.

In the current paper, the number of iterations was set to 500 (360 sec in our case), since this number has been proven to be large enough to obtain the optimal registration results. An example can be seen in Figure 1, which depicts the mean residual displacement (MRD) as a function of iteration time for different study phantoms and B-spline knot spacing (BKS) = 15 mm. After 500 iterations (360 s), an MRD plateau was reached for all phantoms.


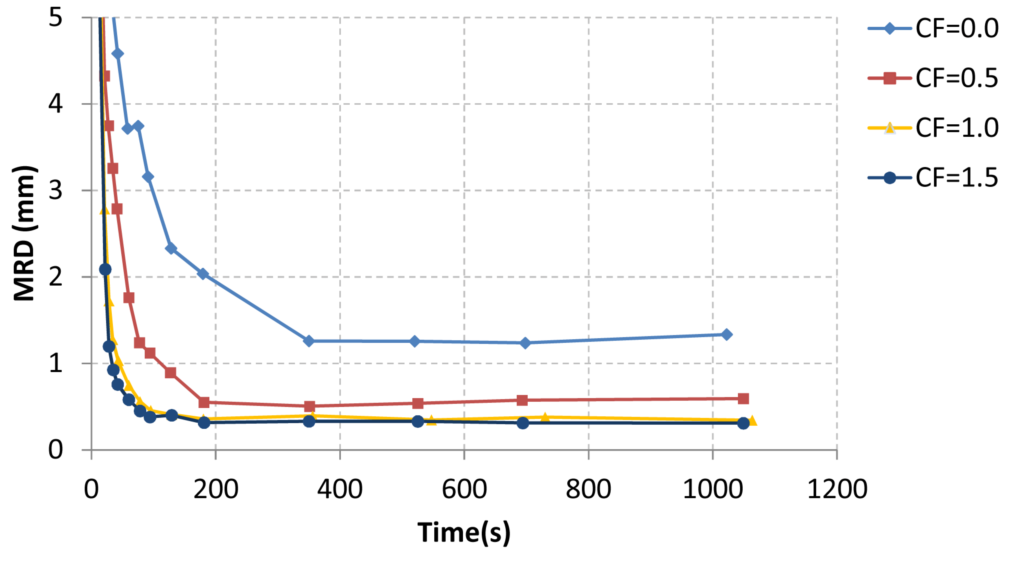


GF=0.0
GF=0.5
GF=1.0
GF=1.5


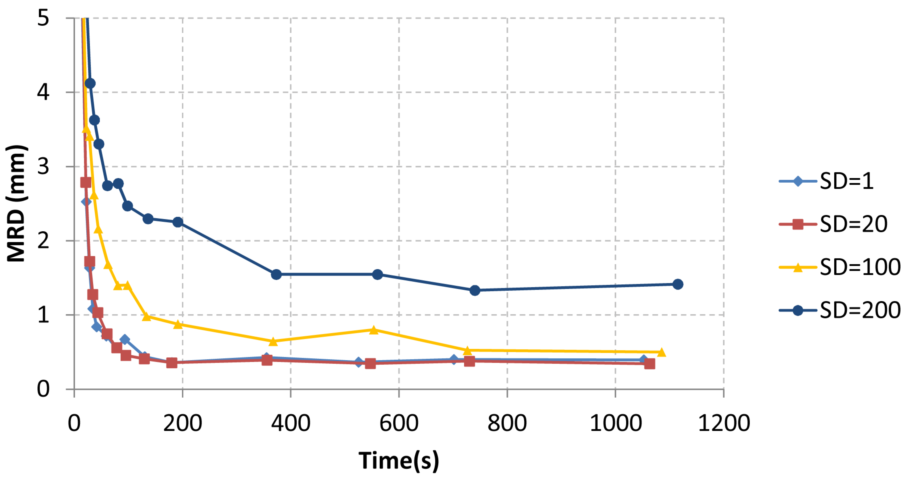


SD=1
SD=20
SD=100
SD=200

Figure 1. Mean residual displacement (MRD) as a function of iteration time, for different phantoms (GF = grey-value factor, SD = 1 standard deviation of the image noise (HU)). 360 seconds corresponds to 500 iterations. B-spline knot spacing was set to 15 mm, SD to 20 HU (upper graph) and GF to 1.0 (lower graph).
